# Development of an evidence-based model for predicting patient, provider, and appointment factors that influence no-shows in a rural healthcare system

**DOI:** 10.1186/s12913-023-09969-5

**Published:** 2023-09-14

**Authors:** Abdul R. Shour, Garrett L. Jones, Ronald Anguzu, Suhail A. Doi, Adedayo A Onitilo

**Affiliations:** 1 Cancer Care and Research Center, Marshfield Clinic Research Institute, Marshfield Clinic Health System, Marshfield, WI USA; 2 Information Technology and Digital Services Analytics, Gundersen Health System, Marshfield, WI USA; 3 Institute for Health and Equity, Medical College of Wisconsin, Milwaukee, WI USA; 4 Department of Population Medicine, College of Medicine, Qatar University, Doha, Qatar

**Keywords:** Appointment no-shows, Evidence-based predictive model, Rural, Healthcare

## Abstract

**Background:**

No-show appointments pose a significant challenge for healthcare providers, particularly in rural areas. In this study, we developed an evidence-based predictive model for patient no-shows at the Marshfield Clinic Health System (MCHS) rural provider network in Wisconsin, with the aim of improving overbooking approaches in outpatient settings and reducing the negative impact of no-shows in our underserved rural patient populations.

**Methods:**

Retrospective data (2021) were obtained from the MCHS scheduling system, which included 1,260,083 total appointments from 263,464 patients, as well as their demographic, appointment, and insurance information. We used descriptive statistics to associate variables with show or no-show status, logistic regression, and random forests utilized, and eXtreme Gradient Boosting (XGBoost) was chosen to develop the final model, determine cut-offs, and evaluate performance. We also used the model to predict future no-shows for appointments from 2022 and onwards.

**Results:**

The no-show rate was 6.0% in both the train and test datasets. The train and test datasets both yielded 5.98. Appointments scheduled further in advance (> 60 days of lead time) had a higher (7.7%) no-show rate. Appointments for patients aged 21–30 had the highest no-show rate (11.8%), and those for patients over 60 years of age had the lowest (2.9%). The model predictions yielded an Area Under Curve (AUC) of 0.84 for the train set and 0.83 for the test set. With the cut-off set to 0.4, the sensitivity was 0.71 and the positive predictive value was 0.18. Model results were used to recommend 1 overbook for every 6 at-risk appointments per provider per day.

**Conclusions:**

Our findings demonstrate the feasibility of developing a predictive model based on administrative data from a predominantly rural healthcare system. Our new model distinguished between show and no-show appointments with high performance, and 1 overbook was advised for every 6 at-risk appointments. This data-driven approach to mitigating the impact of no-shows increases treatment availability in rural areas by overbooking appointment slots on days with an elevated risk of no-shows.

## Background

No-shows occur when a patient fails to appear for a scheduled appointment without prior notification to the healthcare practitioner; failing to attend outpatient visits negatively influences healthcare services, especially in clinics serving medically underserved populations [1–4]. Today, one of the most serious issues confronting health institutions is the presence of patients who fail to show up for their appointments [5]. This affects resource utilization and poses risks to the quality of healthcare services, including the loss of projected revenue, particularly in areas where resources are expensive and in high demand [1, 6]. In fact, an earlier study estimated a $150 billion annual opportunity cost for the United States healthcare industry as a result of no-shows [7]. As a result, reducing patient no-shows is not only crucial for improving clinic performance and promoting efficiency measures, but the need for interventions aimed to target reasons for no-shows in order to reduce no-show rates, enhance access, and reduce health inequalities in underserved patient populations.

The literature highlights a wide range of factors contributing to no-shows in healthcare appointments, particularly in clinics serving underserved populations. These factors encompass patient demographics, clinical information, appointment scheduling details, and historical attendance [4, 8–14]. For instance, studies have identified younger patients, Black or Hispanic patients, and those on Medicaid as more likely to miss appointments, with forgetting and miscommunication as the main reasons [4]. Other significant predictors include the day of the week, appointment lead time, prior no-show history [14], patient age, insurance type [8], socio-demographic characteristics, clinical factors [9], age, sex, marital status, and the number of prior visits [10]. Furthermore, research emphasizes the importance of considering each patient’s attendance history [11] and incorporating time-dependent modeling [12], while also addressing specific patient populations, such as those with diabetes [13].

While the literature highlights several factors contributing to no-shows, previous studies have been undertaken to predict appointment no-shows and develop strategies to mitigate their consequences which have a detrimental influence on healthcare systems [2, 3, 5, 6, 14, 15]. In 2015, Woodward et al. used logistic regression to predict no-shows in patients infected by Human Immunodeficiency Virus. The significance of this work is that characteristics such as the presence of drugs or heterosexual contact were found as significant, although age, which is usually employed in other work, was deemed irrelevant [16]. This conclusion shows that highly informative variables in one situation may not be so in another. The study’s drawback is that no performance metrics were provided. This was not the case with Torres et al., who reported an Area Under Curve (AUC) of 0.71 in their study [17]. Their logistic regression model was built by including just the variables that were significant in the individual models. In 2010, Daggy et al. published one of the first papers in which the estimation of no-show probabilities was incorporated into a scheduling system; about the model’s performance, the authors reported an AUC of 0.82 in a database with a 15.2% no-show rate utilizing a training and test set-up. Using these probabilities, the scheduling system was able to reach a $100 projected benefit per patient [18]. Norris et al. (2014) studied if examining no-shows and cancellations together would enhance no-show predictions, using both multinomial logistic regression and decision trees [19]. Their research revealed that the best results were obtained by utilizing a binary logistic regression that only considered no-shows. This method identified no-shows with an accuracy of 81.5%. This result, however, did not achieve the 91.1% that would be obtained if all patients were categorized as show. The same year, Huang and Hanauer examined the subject from the standpoint of planning systems [14]. They suggested that in no-show prediction, a false positive is a more critical concern than a false negative. When a patient is marked as a no-show when he or she actually attends, the overbooking planning systems suffer greatly. For example, it lengthens the patient’s stay in the clinic and raises the cost of the doctor’s additional time. This fact was considered when determining the threshold. Despite achieving an accuracy of 86.1% in a database with an attendance rate of 88.8%, patients’ waiting time for medical services was cut by 6–8%.

There has been increased interest in decision trees as a means of predicting no-shows, and decision trees are now the most widely utilized tactic after regression models [5]. Aladeemy et al. in 2020 used decision trees to predict no-shows, and evaluated various techniques such as decision trees, random forest, k-nearest neighbors, support vector machines, boosting, naive Bayes, and deep learning [20]. They demonstrated that decision trees produced the best results. Lotfi and Torres employed multiple algorithms in 2014 to create decision trees that forecast no-shows in the most recent appointment [21]. Chi-squared Automatic Interaction Detector (CHAID), Exhaustive CHAID, Classification and Regression Trees, and Quick, Unbiased, Efficient Statistical Tree were among the techniques evaluated. The best model had an accuracy of 78%, which was lower than the attendance rate of 84%. Glowacka et al. demonstrated that including estimated probabilities into a scheduling system increased center utilization from 46 to 72.9% [22]. For the first time, Lee et al. employed Gradient Boosting (GB) to aggregate forecasts from multiple decision trees in 2017 [23]. They reported an AUC of 0.83 using 60 variables obtained by text mining and several socio-demographic characteristics. Elvira et al. used GB the following year to mitigate the problem of class imbalance [24]. However, their findings were limited because the AUC was less than 0.75. The authors found that they did not have enough information to reliably predict missed appointments. Srinivas and Ravindran then created a stacking model to predict no-shows in a primary care center, which included predictions from neural networks, random forest, and stochastic GB, achieving an AUC of 0.85 [25].

Our study aims to build upon existing research by developing a predictive model specifically tailored to rural healthcare environments, where access and resources are uniquely challenging. However, we acknowledge the potential of our model and overbooking scheme for broader application, as demonstrated in previous studies. Huang and Hanauer (2014) developed an evidence-based predictive model using a decade’s worth of data from a pediatric clinic. They found that their overbooking approach, similar to ours, led to a significant reduction in patient waiting time, overtime, and total costs. Although their study focused on a pediatric clinic, the underlying principles of their model are generalizable and could be applicable to other healthcare settings, supporting the potential for our model to extend beyond rural healthcare networks [14]. Daghistani et al. (2020) used outpatient visit data and machine learning to identify factors influencing no-show rates. They achieved high accuracy and Receiver Operating Characteristic (ROC) curve scores, indicating the effectiveness of machine learning approaches to predict patient no-shows [2]. This further suggests that the predictive model we have developed may also be effective in different healthcare contexts. Additionally, a study by Benedito Zattar da Silva et al. (2022) investigated no-shows in a radiology department of a Brazilian hospital and concluded the importance of utilizing machine learning models to develop strategies to minimize patient no-shows [6]. Their study, although based in a radiology department, highlights the versatility of machine learning models in predicting patient no-shows across a variety of healthcare settings.

In this study, we developed an evidence-based predictive model for patient no-shows at the Marshfield Clinic Health System (MCHS) rural provider network in Wisconsin, with the aim of improving overbooking approaches in outpatient settings and reducing the negative impact of no-shows in our underserved rural patient populations. Our objectives are to estimate appointment no-show probabilities, enhance overbooking approaches, improve clinic performance and promote efficiency strategies, maximize clinic capacity utilization, and minimize patient waiting time and clinic overtime expenditures, based on the potential impact of our model and its high-performance results.

## Methods

### Clinical environment

As an integrated rural health system, MCHS aims to improve people’s lives by providing accessible, affordable, and compassionate health care. With over 1,600 providers representing 170 specialties, a health plan, and research and education programs, our Health System covers Wisconsin and Michigan’s Upper Peninsula. Our Clinic has over 60 locations, 11 hospitals, Marshfield Children’s Hospital, Marshfield Clinic Research Institute, Security Health Plan, and the MCHS Foundation. We have four Joint Commission-accredited outpatient surgery centers that perform over 2,500 different types of outpatient surgical procedures, including ear, nose, and throat surgery, gastroenterology, general surgery, neurosurgery, obstetrics and gynecology, and ophthalmology. Our Clinic was founded in 1916 under Wisconsin law and operates as a charitable corporation with all of its assets held in a charitable trust. Our clinic is one of only a handful of large autonomous nonprofit medical clinics in the United States. Our patients have access to various physicians and other medical professionals. Neuro-oncology, maternal-fetal medicine, pediatric orthopedic surgery, and electrophysiology are a few examples of specializations.

A patient can make an appointment with our providers in three ways. First, contact (call) care teams or the doctor’s office if the patient already has a doctor to discuss appointment options. Second, online appointments via the patient portal. Patients can submit an appointment request using an online form and provide their first and last names, address, date of birth, daytime contact number, appointment preferences (specialty, and location), and reason for appointment (optional). Third, in circumstances where the patient is unsure whom to contact, we provide a phone number for patients to contact the resource information coordinator, who will help patients connect with a member of the care team or find the necessary information.

### No-show data and modeling

We introduced a process to reduce the impact of no-shows by developing a no-show predictive model. Our model uses a set of variables to predict the likelihood of a patient not showing up for a given appointment. Retrospective (2021) appointment data was obtained from the MCHS scheduling system, which included a population sample of 1,260,083 (N) total appointments from 263,464 patients, as well as their demographic, appointment, and insurance information. Our machine-learning model was trained and validated on MCHS ambulatory appointments. Every weekday, the model scores every ambulatory appointment in a prospective rolling 2-week window and generates a report. Our model assigns a score between 0 and 1 to each ambulatory appointment, and a cut-off threshold was chosen to indicate that the appointment is ‘at risk’ of being a no-show. Based on the model’s determination of at risk, we can make a recommendation to overbook 0, 1, or 2 schedule slots per provider per day (maximum of 2 overbooks).

Our model uses a set of variables to predict the likelihood of appointment no-shows. To develop the model, we collected appointments and associated data (features) from the MCHS data warehouse such as demographic factors, insurance information, appointment characteristics, appointment history, and provider characteristics. We collected *demographic factors* including the sex of the patient, oral language, age, ethnicity, 5-digit zip code for the patient, straight line distance from the center of the patient’s zip code to the facility where the appointment took place, and county, household characteristics, racial and ethnic minority status, housing type and transportation (linked from the Social Vulnerability Index developed by the Centers for Disease Control and Prevention). *Insurance information* included insurance type codes associated with the patient’s appointments in the past year. *Appointment characteristics* included the month of the appointment start date, day of week of the appointment start date, the hour of the appointment start time, duration of the appointment in minutes, number of days between the appointment creation date and the appointment start date, appointment type, and campus where the appointment took place. *Appointment history* included the number of appointments shows for the patient in the year prior to the appointment date, the number of appointment no-shows for the patient for a year prior to the appointment date, the total number of appointments for the patient in the same date as the appointment, and whether or not the patient confirmed the appointment. *Provider characteristics* were the provider’s department, specialty type, primary facility, and provider type.

### Feature preprocessing

We used descriptive statistics to explore the impact of variables on show or no-show status. Before calculating predictions, the features were preprocessed by the model. We explored logistic regression, random forests, and eXtreme Gradient Boosting (XGBoost) based models and found XGBoost to perform the best.

**Fig. 1:**
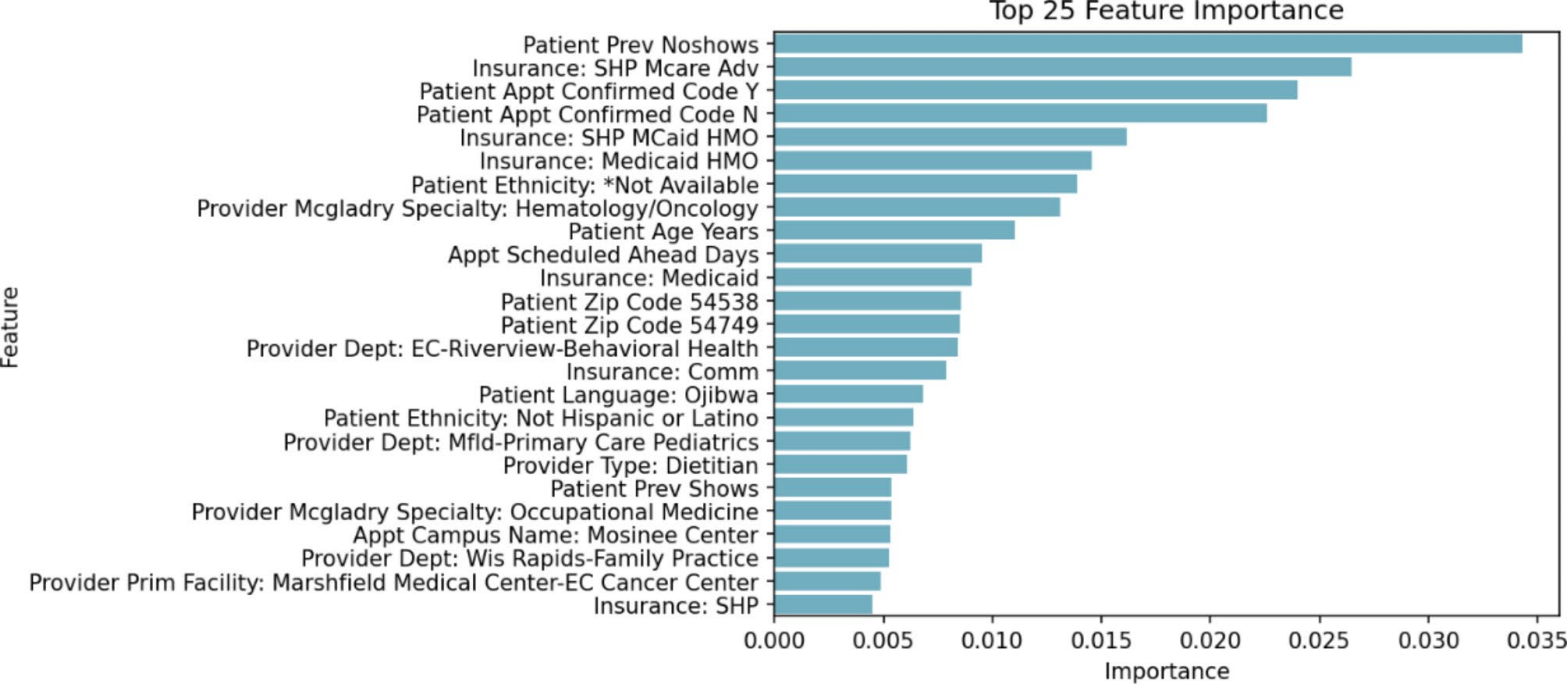
Feature Importance

**Fig. 2:**
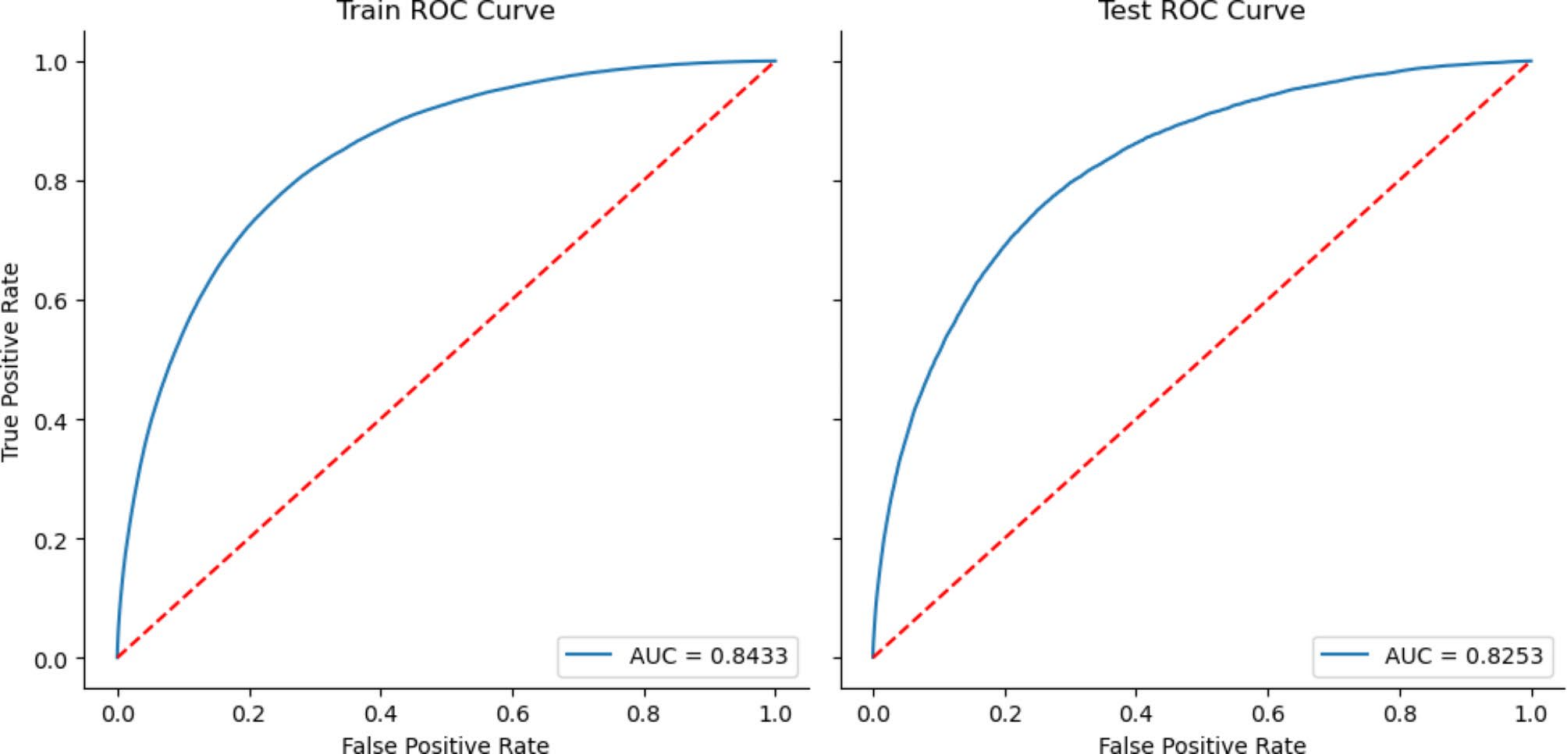
Performance. Area under the ROC (AUC): 0.8433 with train and 0.8253 with test

We chose the XGBoost algorithm for our study after comparing its performance with other machine-learning models, such as logistic regression and random forests. Our selection of XGBoost was influenced by several factors. First, XGBoost can determine feature importance, which we employed in our study to identify the most significant predictors of patient no-shows (Fig. 1). The XGBoost model yielded the best performance metrics in our tests, achieving an Area Under the Curve (AUC) of 0.84 for the training dataset and 0.83 for the test dataset (Fig. 2). This performance surpassed the results obtained from logistic regression and random forests. While logistic regression is simpler and more interpretable, it could not effectively capture the complex non-linear relationships present in our data as well as XGBoost. This limitation is a known characteristic of linear models like logistic regression [26]. In contrast, random forests are a powerful tool for handling non-linear relationships and variable interactions. However, they can create more complex models and overfit the training data, potentially impairing the model’s performance on unseen data [27]. XGBoost, a type of gradient boosting, constructs a robust predictive model by assembling weaker prediction models, typically decision trees. It has demonstrated high predictive accuracy and the ability to effectively manage various data types, missing values, and outliers [28]. It is important to note that no model is inherently superior in every situation; model selection should always be guided by the specific data and problem being addressed. In our case, XGBoost offered the optimal balance of predictive power, robustness, and interpretability.

Our model used various types of features, including interval, count, and categorical features. For interval features, we imputed missing values with the mean of the values in the training set using appointment and demographic characteristics. For count features, we imputed missing values to 0 for patients’ appointment history and insurance information. For categorical features, we imputed missing values to a “Missing” category, and the values were then one-hot-encoded. This preprocessing step ensured that our model could efficiently utilize the available data and minimize the impact of missing values on its performance.

### Data cleaning

In addition to handling missing values, we performed other data cleaning steps to ensure the quality of the data used in the model. These steps included checking for data entry errors, inconsistencies, and duplicates, and correcting them as necessary. Regarding outliers, we examined the data distribution for each feature and assessed their potential impact on model performance. In cases where outliers were present, we applied robust techniques, such as winsorization or log transformations, to mitigate their influence on the model. By carefully cleaning the data and addressing outliers, we ensured that our predictive model could accurately capture the patterns in the data and provide reliable recommendations for overbooking.

## Results

### Factors driving no-show rate

Figure 1 demonstrates that several factors play a crucial role in predicting patient no-shows. The most significant predictor is the patient’s previous no-shows. By calculating the average information gain across all decision tree splits in the model, we determined feature importance. Among the 25 factors, the top three features were the patient’s previous no-shows (0.35), Medicare Insurance Security Health Plan (SHP) advantage (0.26), and appointment confirmation (yes) (0.25).

Table 1 displays the descriptive statistics for 1,260,083 appointments. The no-show rate in both the train and test datasets was 6.00%, with 60,265 no-shows and 947,801 shows in the training set, and 15,066 no-shows and 236,951 shows in the test set. The no-show rate varied depending on lead time and age. Appointments with a lead time of over 60 days had the highest no-show rate (7.73%), followed by 31–60 days (7.41%), 16–30 days (6.10%), and 0–15 days (4.31%). For the lead time bins in Table 1, we initially utilized bins of 15 days to capture the differences in no-show rates within shorter time frames, which are more likely to exhibit greater variability. In contrast, for lead times over 30 days, we employed a bin of 30 days, as longer lead times generally exhibit less variability in no-show rates, and a larger bin effectively captures the overall trend in the data. Younger patients had higher no-show rates than older patients. The highest no-show rate was observed among patients aged 21–30 years (11.84%), followed by 31–40 years (10.34%), 11–20 years (9.34%), 41–50 years (9.06%), 0–10 years (8.52%), and 51–60 years (6.37%). Patients over 60 years of age had the lowest no-show rate at 2.86%.

**Table 1:** Descriptive Statistics (N = 1,260,083 visits)

	No-show	Show	No-show Rate
**Data set**			
Train	60,265	947,801	6.00
Test	15,066	236,951	6.00
**Lead Time(days**)			
0–15	23,076	512,111	4.31
16–30	12,258	188,575	6.10
31–60	12,251	153,052	7.41
Over 60	27,746	331,014	7.73
**Age (years)**			
0–10	7,085	76,118	8.52
11–20	9,297	90,200	9.34
21–30	10,290	76,613	11.84
31–40	10,720	92,985	10.34
41–50	10,054	100,930	9.06
51–60	10,374	152,458	6.37
Over 60	17,511	595,448	2.86

### Figure 3: prediction distributions

**Fig. 3:**
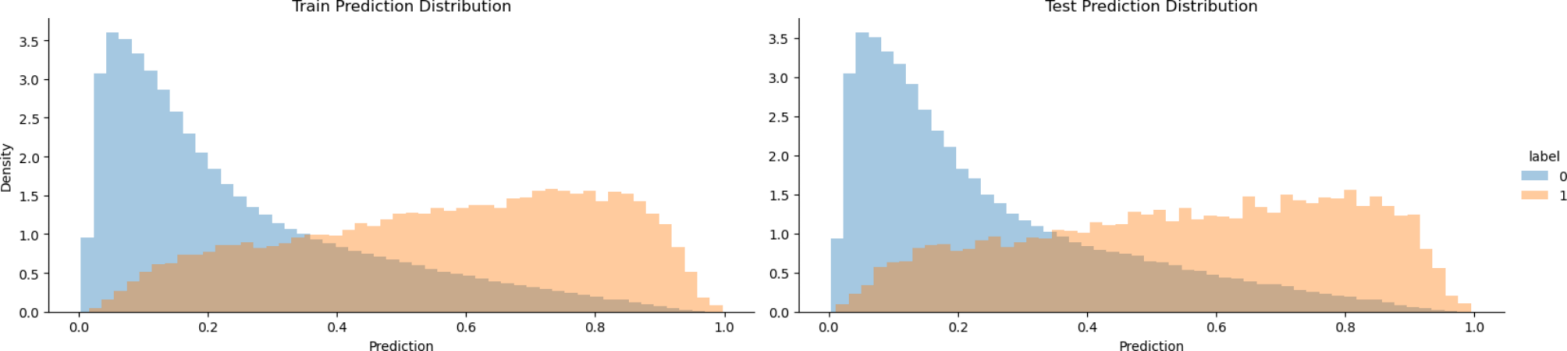
Prediction Distributions (0 = Show, 1 = No-Show)

In Fig. 3, the model score distribution plots for both the training and prediction datasets are presented. The blue distribution represents true show appointments (0), while the orange distribution signifies true no-show appointments [1]. The model score is plotted on the x-axis, with higher scores indicating a higher likelihood of a no-show. We normalized the height of the two distributions to a density function rather than showing the count, as there are fewer no-show appointments than show appointments. The graphic demonstrates that the model can effectively distinguish between show and no-show appointments, with the distribution of model scores for actual no-show appointments centered near the lower end of the scores and the distribution for show appointments centered toward the upper end of the scores.

The AUC for the training dataset was 0.84, while the AUC for the test dataset was 0.83. By illustrating the close proximity of the AUC values, we emphasize the consistency of the model’s performance across both datasets.

### Figure 4: Cut-off Points

**Fig. 4:**
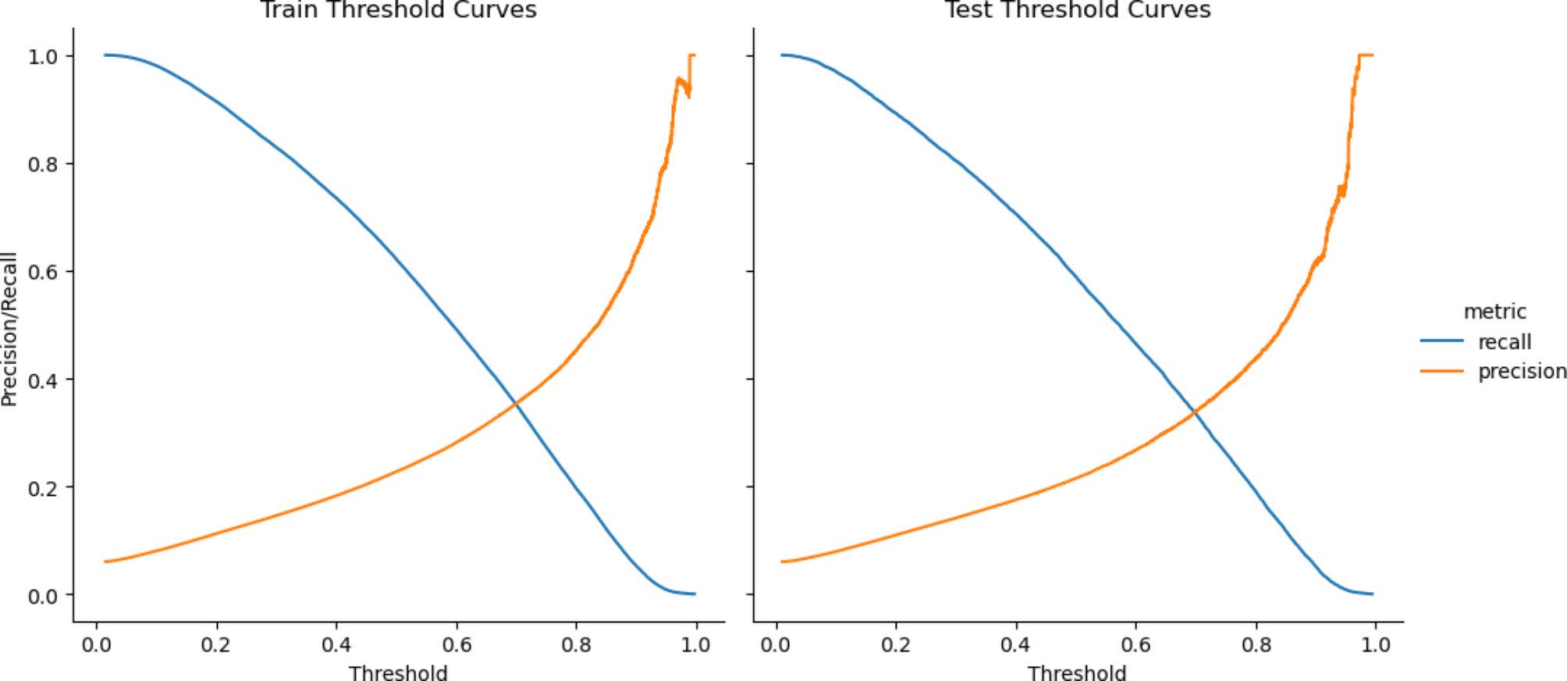
Cut-off points. Cut-off points curve to give a more concrete idea about performance. Focus on the confusion matrix numbers, sensitivity and positive predictive value

For Fig. 4, we identified cut-off points curve to provide a more concrete idea about the model’s performance. The figure focuses on the confusion matrix numbers, sensitivity, and positive predictive value, highlighting the optimal cut-off point for the prediction of patient no-shows. Figure 4 is necessary as it offers a visual representation of the model’s performance, allowing readers to understand the balance between sensitivity and positive predictive value.

Table 2 displays the performance of our predictive model using a 0.4 cut-off, showcasing the model’s recall (sensitivity) and positive predictive value. High-risk patients were identified based on a cut-off value of 0.40, which was determined in consultation with business subject experts and by considering the model’s performance. Based on these results, we recommended overbooking one appointment for every six at-risk appointments. The recommendation stemmed from the positive predictive value of approximately 17% for appointments identified as high risk. In consultation with business subject experts and taking into account their current workflow and the model’s performance, we decided to overbook one patient for every six high-risk patients (with a maximum of two overbookings). The cut-off value was set at 0.40, the sensitivity was 0.71, the precision (positive predictive) value was 0.18, and one overbooking was recommended for every six at-risk appointments. The workgroup determined the cut-off based on the positive predictive value for high-risk appointments, which led to the overbooking recommendation. The cut-off and maximum of two overbookings were established through discussions with business subject experts, ensuring they were based on practical considerations rather than being arbitrarily chosen by the model.

**Table 2:** Performance: 0.4 Cut-off

Model		Actual	
		Negative	Positive
**Predicted**	Negative	186,746	4427
	Positive	50,205	10,639
Cut Off: 0.4 Recall/Sensitivity: 0.706 Positive Predictive value: 0.175			
1 overbook for every 6 at-risk appointments			

## Discussion

Our study addressed two primary questions: (1) Can we predict the likelihood of rural clinic no-shows based on demographic and other characteristics? and (2) Can we use this likelihood to characterize the prospective patient risk of no-shows and employ that characterization to enhance clinic performance? To answer these questions, we developed a predictive model utilizing demographic, appointment, and insurance data to estimate patient no-shows in rural healthcare clinics. The most significant factors in predicting patient no-shows were patient’s previous no-shows, Medicare SHP advantage, and appointment confirmation. The final model, XGBoost, exhibited high performance in distinguishing show and no-show appointments, achieving an AUC of 0.84 for the train set and 0.83 for the test set. Patients with longer appointment wait times (over 60 days) and younger patients (aged 21–30) were found to have higher no-show rates. Leveraging the predictive model to assess patients’ no-show risk, we proposed an overbooking strategy to optimize clinic performance, recommending overbooking one appointment for every six at-risk appointments per provider per day. This data-driven approach enhances treatment availability in rural areas by efficiently allocating appointment slots, mitigating the impact of no-shows, and maximizing clinic resource utilization.

Our study builds upon existing research by developing a predictive model specifically tailored to rural healthcare environments, where access and resources are uniquely challenging. Firstly, many existing studies indicate that previous no-shows, insurance type, and appointment confirmations are crucial predictors of no-shows, but the emphasis on these factors tends to vary based on the settings studied [29–31]. In urban areas, studies have found that socioeconomic factors and access to transport also play significant roles [14, 32, 33]. However, such factors may have less bearing in rural settings, where healthcare resources and access are more constrained [34, 35]. The Medicare SHP advantage, in particular, stands out in our study because rural populations tend to have higher Medicare enrollment rates [34]. Additionally, rural healthcare systems may use appointment confirmation systems more extensively, given their tighter resource constraints and higher stakes in preventing no-shows [36, 37]. Secondly, in terms of the uniqueness of our study, our findings contribute to the literature by identifying the relative importance of various factors in predicting no-shows in rural healthcare environments. Although previous no-shows and appointment confirmations have been recognized as important predictors in both rural and non-rural settings [38, 39], our study underscores their heightened significance in rural contexts. This may be attributable to the unique challenges faced by rural patients, such as longer travel distances and limited access to healthcare facilities, which could amplify the impact of prior no-show behaviors and appointment confirmations [35]. Thirdly, some factors deemed important in non-rural studies may be less impactful in our rural context due to the specific characteristics of rural healthcare. For instance, access to transportation, a common predictor in urban environments, may be less influential in rural settings where patients are accustomed to traveling long distances to receive care [32]. Lastly, it is essential to highlight the differences between our study and those conducted in non-rural settings. Rural healthcare environments are often more resource-constrained and suffer from limited access to services. The crucial predictors identified in our study – previous no-shows, Medicare SHP advantage, and appointment confirmations – can help tailor interventions specifically for these environments, thereby enhancing their effectiveness and resource efficiency.

Moreover, we diverge from traditional methodologies by introducing a dynamic, patient-specific overbooking strategy, based on a machine learning model, to predict and manage patient no-shows, thereby addressing the inefficiencies inherent in static overbooking strategies previously discussed in the literature. Previous studies have mainly explored static overbooking strategies where a fixed number of slots are overbooked regardless of the patients’ characteristics or the clinic context [40–42]. Conversely, our scheme is dynamic, utilizing a machine learning model to predict the likelihood of no-shows based on individual patient characteristics and appointment factors. This allows for an adaptive overbooking strategy, which can optimize clinic utilization more effectively by accounting for varying no-show probabilities. In our scheme, we overbook one appointment for every six predicted at-risk appointments, rather than applying a standard ratio irrespective of no-show risk. This patient-specific, data-driven approach was based on an empirical cut-off derived from the model’s predictive accuracy and agreed upon with business subject matter experts. The strategy aims to balance the risk of long waiting times if patients attend their appointment against the wasted resources when patients do not show up. Furthermore, our method is flexible, and the parameters can be adjusted according to the context and priorities of the individual clinic. Contrary to a static approach, our model considers both the potential benefit of serving more patients and the operational risk of overbooking in the healthcare context. Thus, our scheme’s contribution lies in its adaptability, individualized approach, and data-driven nature, which outperforms and adds a significant contribution to the traditional overbooking strategies widely discussed in the literature [43, 44]. We plan to collect more data in the future to continue validating and refining our model.

Our study is based on a large Electronic Health Record (EHR) dataset with demographic, appointment, and insurance information commonly found in most health systems enhancing our model’s applicability across various settings [45]. Consistent with the literature, the predictors used, such as age, appointment wait times, and insurance type, are significant factors associated with no-shows [30, 46], suggesting our findings’ relevance in other contexts. Our model, developed and evaluated using three widely-used machine learning techniques (logistic regression, random forests, and XGBoost), can be applied to other health systems with similar data [47, 48]. The robustness of our model is demonstrated by AUC values greater than 80% for both train and test datasets. The overbooking scheme, recommending 1 overbook for every 6 at-risk appointments, is derived from a data-driven approach, considering benefits and risks, and can be adapted to other health systems by adjusting parameters based on context and priorities. To further bolster generalizability, we suggest conducting a multi-center study involving diverse health systems and patient populations, enabling model validation on external datasets.

In rural communities, healthcare access is often impeded by challenges such as limited provider availability, and extended travel distances, which can influence appointment adherence [34, 49]. Consistent with prior research [30, 46], our descriptive analysis found that patients with longer appointment wait times (over 60 days) and those in the younger age group [21–30] had higher appointment no-show rates. The rural context may exacerbate these factors, with fewer healthcare providers leading to increased wait times, and younger patients potentially facing challenges such as limited transportation options or work schedule conflicts [34, 49]. Recognizing these factors and addressing them through targeted interventions may improve appointment adherence and healthcare access in rural communities.

The overbooking technique we proposed, recommending one overbook for every six at-risk appointments per provider per day, is based on the model’s predictions, with a cut-off set at 0.4, sensitivity of 0.71, and positive predictive value of 0.18. This cut-off was determined by the workgroup and discussed with business subject experts to ensure its compatibility with their current workflow. Comparatively, standard overbooking methods often involve a fixed number of overbooked appointments regardless of patient risk, which may lead to increased wait times, patient dissatisfaction, and inefficiencies in resource allocation [14]. Our approach, tailored to rural healthcare environments, takes into account the unique challenges faced in these settings, such as limited access to care and resources. By focusing on at-risk appointments and incorporating logistic regression, random forests, and XGBoost techniques, our model offers a more targeted overbooking strategy that maximizes resource utilization while minimizing potential disruptions.

Our team, which comprised subject matter experts from the business sector, engaged in a meticulous dialogue when settling on an appropriate threshold point. A primary point of discussion during this process was striking a balance between the model’s sensitivity, its positive predictive value, and the operational implications for healthcare providers. This threshold delivers a strong equilibrium, ensuring sufficient sensitivity while managing the volume of false positives. Additionally, we proposed an overbooking strategy, suggesting an extra appointment booking for every six appointments considered at-risk, based on the 17% positive predictive value for appointments categorized as high-risk. This approach considers both the current workflow of healthcare providers and the predictive model’s performance. To maintain feasible clinic operations, we set a maximum of two overbookings. We did contemplate other potential cutoffs during our deliberations. However, we chose the 0.40 cutoff as it offered the most efficient compromise between model efficacy and pragmatic factors, such as workflow management and overbooking restrictions.

### Strengths and limitations

One of our study’s primary strengths was that it used a large sample size (N = 1,260,083 appointments) from the rural hospital database. The extensive dataset allows for even more samples to be separated into data sets for predictive model training, testing, and validation. Our model simplifies operations by relying on administrative data and basic patient demographic data received by the call center during appointment bookings, even for new patients. Our work demonstrates that using machine learning techniques, such as XGBoost, can develop a strong performance prediction model that is efficient for clinical processes. XGBoost was found to be superior to logistic regression and random forests, which were also explored in our study. However, a drawback of our study is that we did not include patients’ socioeconomic status, level of education, and medical conditions, which can be significant contributors to no-shows [5]. Also, our research reports the findings of an automated machine learning binary classifier method used to identify non-showers versus showers on several variables. We did not create a patient score to be used to evaluate the best interval groups when making a prediction rule over a binary outcome using a logistic regression model. The interval likelihood ratio would have been computed and interpreted in terms of posterior probability of no-shows, which the medical community would have found more intelligible. Future studies should address this issue because a unique patient score and how it is calculated may offer a sense of the patient rather than what has only been done.

Finally, we acknowledge that our overbooking recommendation, which involves scheduling one extra patient for every six high-risk patients, might seem conservative. Nevertheless, the intention is to balance addressing no-shows and minimizing potential overbooking consequences, such as overcrowding and extended wait times. Our proposed overbooking strategy is designed to enhance appointment availability and utilization without excessively burdening healthcare providers and patients. The decision to overbook one patient for every six high-risk patients is based on the model’s positive predictive value of 0.18, which indicates that approximately 1 in 6 predicted high-risk no-shows will actually not attend their appointment. While we recognize that this could result in occasional overlaps when the 6th high-risk patient attends their appointment, spreading overbooking across a week should help mitigate the overall impact. Although the no-show rate of 6% is relatively low, we believe that even minor improvements in appointment utilization can positively affect healthcare delivery, especially in rural areas with limited resources. Implementing a predictive model like the one we developed could assist in optimizing scheduling practices and enhancing patient access to care. In light of these concerns, we emphasize the need for future research to evaluate the real-world impact of various overbooking strategies based on our model’s predictions.

### Study implications

Despite these limitations, our study provides a method for forecasting clinic no-shows that makes use of various data variables that are expected to be available at most health centers and clinics with an EHR and a scheduling system. Our suggested overbooking technique offers an alternate method of proactively overbooking patients by considering a particular appointments’ likelihood of a no-show based on provider and patient data, including prior no-show history. Moreover, given the likelihood that the data elements used in our predictive model will be readily available, it is important to think about how such a model might be put into practice and applied to the routine activities of a clinic, where staff members in charge of scheduling should quickly decide whether to overbook patients. There is no reason why such technology could not be added to EHR and scheduling systems [14], even if we are not aware of any that already integrate prediction algorithms for no-shows in rural health systems. In such a case, the scheduling system may offer forecasts that would direct schedulers to the ideal window of time for an overbook, if that were to be required. Additionally, the system might routinely update its model automatically to include new data and results. It is worth noting that our study established a prediction model for several practices rather of a single practice, as the was the case in previous analysis [14]. Having numerous specialties with a diverse patient mix aided in the development of a model that attained optimal performance, and our model offers advantages over existing overbooking approaches. However, the variables we chose for our model were selected because they were readily available, but there are likely many other variables that contribute to no-shows that are more difficult to quantify and may be difficult to collect on a routine basis for all patients, such as chronic diseases and area-level social determinants of health.

At the contextual level, rural health care providers have expressed how difficult it can be to look up and prepare for patients who do not show up. For the month of December 2022, for example, MCHS had a 10% no-show rate (which was higher than the national benchmarking of 5%), which was not captured in our current analysis, which reported a 6% no-show rate. The problem is certainly multifactorial and our study findings will help to solve it by predicting patients who do not show up and which factors to focus on to reduce the no-show rate. While no solution is perfect, we can always improve this process at the contextual level as we go. Other regions have been working on identifying no-shows using algorithmic models that can predict which days we may have a high number of no-shows. We would like to take this approach at MCHS, with the caveat that any overbooked time will be discussed first between the Operations Manager and the provider. This first approach, we believe, will be beneficial to us as well in terms of access. The second approach is to identify those patients and create a model in which a phone call, second reminder, or other action is taken to prevent the no-show in the first place. To identify these patients, we are already collaborating with the MCHS information technology office. Ultimately, the most interesting part of this effort would be the impact of the model on no-shows and patient care.

We wish to highlight the successful implementation of our predictive model and the accompanying overbooking policy at MCHS. Our model is actively used to identify high-risk ambulatory appointments, guiding our recommendations for overbooking 0, 1, or 2 schedule slots per provider per day, with a cap set at 2 overbookings. While these are recent developments, initial feedback from healthcare providers and administrators has been broadly positive, suggesting that the model assists in streamlining workflows and managing no-show occurrences more effectively. However, we acknowledge the need for a more extensive evaluation of the long-term effects on staff acceptance, revenue generation, patient satisfaction, and waiting times. Our future publications will address these aspects based on ongoing data monitoring. Importantly, the model was designed in close collaboration with business subject experts, ensuring a balanced approach between sensitivity, positive predictive value, and practical workflow considerations. A key outcome of this collaboration is our overbooking policy, which recommends one overbooked appointment for every six high-risk appointments based on a positive predictive value of approximately 17% for such appointments. This approach ensures manageable clinic operations while effectively reducing the impact of no-shows.

## Conclusions

Appointment no-shows are a major source of concern in rural healthcare systems, and we created an evidence-based predictive model for patient no-shows to alleviate this concern. Using data from a rural scheduling system, which included 1,260,083 appointments, we provided descriptive statistics to relate variables with show or no-show status, and logistic regression, random forests, and XGBoost were used to develop a no-show prediction model, determine cut-offs, and assess model performance. We discovered that the no-show rate was 6%. Patients with longer appointments (> 60 days in advance) and those aged 21–30 had the highest no-show rate. The new model distinguished between show and no-show appointments with high performance, and 1 overbook was advised for every 6 at-risk appointments. Our research proved the feasibility of constructing a prediction model based on administrative data gathered on a regular basis and made available in a rural healthcare system to classify patients based on their likelihood of skipping appointments. We exhibited a data-driven approach to maximizing clinic resource use in order to boost treatment availability in remote places by overbooking slots to be filled by patients at high risk of no-shows.

## Data Availability

The data were not made public because this healthcare system did not want to reveal corporate policies that were not the subject of this study. Nonetheless, the data flow charts and codes used to develop the model are available upon request from the corresponding author. All data were also presented in the main manuscript, tables, and figures. All relevant data are within the manuscript and its Supporting Information files.

## Declarations

### Reporting experiments on humans/human data

The analyses were conducted in compliance with applicable guidelines and regulations.
